# Peroneus Longus Tendon vs Hamstring Tendon Autograft for Anterior Cruciate Ligament Reconstruction: A Comparative Study of Functional Outcomes and Graft Characteristics

**DOI:** 10.7759/cureus.112776

**Published:** 2026-07-16

**Authors:** Chetan Tamta, Atul Agrawal, Faiz Akbar Siddiqui

**Affiliations:** 1 Department of Orthopedics, Himalayan Institute of Medical Sciences, Dehradun, IND

**Keywords:** acl reconstruction, anterior cruciate ligament, hamstring tendon, lysholm score, peroneus longus tendon

## Abstract

Background

Anterior cruciate ligament (ACL) injury is a common orthopedic condition, particularly among young and physically active individuals. ACL reconstruction (ACLR) is widely performed to restore knee stability and improve functional outcomes. Hamstring tendon autografts are commonly used for reconstruction; however, concerns regarding inadequate graft diameter, delayed graft incorporation, and donor site morbidity have led to increasing interest in the peroneus longus tendon as an alternative graft source. The present study compared the functional outcomes and graft characteristics of hamstring tendon and peroneus longus tendon autografts in ACLR.

Methods

This observational comparative study was conducted in the Department of Orthopedics at Himalayan Institute of Medical Sciences, Dehradun, over a period of 12 months. A total of 80 patients with ACL rupture undergoing primary ACLR were included and divided equally into two groups. Group A included 40 patients reconstructed using hamstring tendon autografts, while Group B included 40 patients reconstructed using peroneus longus tendon autografts. Baseline demographic and clinical characteristics were recorded preoperatively. Functional outcomes were assessed using the Lysholm Knee Scoring Scale preoperatively and at six weeks and 12 weeks postoperatively. Final graft length and diameter were also compared between the groups.

Results

The mean age of patients was comparable between the hamstring and peroneus groups (30.35 ± 8.58 years vs 30.33 ± 9.35 years; p = 0.990). Male predominance was observed in both groups. The peroneus longus group demonstrated significantly greater graft length (8.37 ± 0.54 cm vs 8.00 ± 0.58 cm; p = 0.004) and graft diameter (7.73 ± 0.57 mm vs 7.40 ± 0.46 mm; p = 0.005) compared to the hamstring group. Preoperative Lysholm scores were comparable between groups (p = 0.410). However, significantly higher postoperative Lysholm scores were observed in the peroneus group at both six weeks (80.70 ± 5.98 vs 74.90 ± 8.11; p = 0.001) and 12 weeks (90.25 ± 3.46 vs 84.57 ± 4.97; p = 0.001). At 12-week follow-up, excellent functional outcomes were achieved in 40.0% of patients in the peroneus group compared to 7.5% in the hamstring group. Postoperative complications were minimal and comparable in both groups, with no cases of graft failure observed during the 12-week follow-up.

Conclusions

Peroneus longus tendon autograft demonstrated significantly greater graft dimensions and superior short-term functional outcomes compared with hamstring tendon autograft in ACLR. Both grafts were associated with low complication rates. The peroneus longus tendon may be considered a safe and effective alternative graft option for ACLR.

## Introduction

Anterior cruciate ligament (ACL) rupture is one of the most common orthopedic injuries, particularly among athletes and physically active individuals. The ACL plays an essential role in maintaining knee stability by preventing anterior translation of the tibia and controlling rotational movements during activities such as running, pivoting, and sudden directional changes [1]. ACL injury can result in instability, impaired function, and reduced quality of life. If left untreated, it may also predispose patients to secondary meniscal injury, cartilage damage, and early osteoarthritis [2]. As a result, ACL reconstruction (ACLR) has become a standard surgical procedure aimed at restoring knee stability, improving function, and enabling a return to preinjury activity levels [3].

Several autograft options are available for ACLR, including bone-patellar tendon-bone, hamstring tendon, and quadriceps tendon grafts [4]. Each graft type has its own advantages and limitations related to biomechanical strength, graft availability, donor site morbidity, and postoperative recovery [5]. Among these, hamstring tendon autografts remain one of the most commonly used grafts worldwide because of their favorable biomechanical properties, ease of harvest, and lower donor site complications compared with patellar tendon grafts [6]. However, hamstring grafts may be associated with reduced hamstring strength, delayed graft integration, and inadequate graft diameter in some patients [7].

In recent years, the peroneus longus tendon has emerged as a promising alternative graft for ACLR. Biomechanical studies have shown that the peroneus longus tendon possesses adequate tensile strength and graft size comparable to hamstring tendons [8]. Potential advantages include preservation of hamstring muscle function, sufficient graft material, and minimal donor site morbidity. Some studies also suggest that preservation of hamstring integrity may contribute to better postoperative muscle performance and dynamic knee stabilization. Nevertheless, concerns remain regarding possible ankle weakness or instability following graft harvest, especially in athletes and individuals who depend heavily on ankle function [9]. In addition, long-term clinical evidence regarding functional outcomes and donor site complications remains limited, and harvesting the tendon requires technical expertise [10].

The incidence of ACL injuries is highest among young adults aged 20-24 years, although increasing rates have also been reported in older active populations [11]. Female athletes are particularly vulnerable to sports-related ACL tears because of hormonal, anatomical, and neuromuscular factors [12]. Globally, ACLR procedures continue to rise, reflecting increasing participation in sports and recreational activities [13,14]. Clinically, ACL tears may present with pain, swelling, instability, and an audible “popping” sound at the time of injury. Physical examination tests such as the Lachman and anterior drawer tests remain important diagnostic tools, while MRI is considered the gold standard for confirming ACL injury and identifying associated intra-articular lesions [15].

Graft selection is one of the most important factors influencing the outcome of ACLR. An ideal graft should provide sufficient biomechanical strength, rapid incorporation, minimal donor site morbidity, and good long-term functional outcomes. Although hamstring tendon grafts are widely accepted, the increasing interest in peroneus longus tendon autografts has created the need for comparative clinical evaluation. Existing literature suggests favorable outcomes with both grafts, but differences in knee stability, muscle strength, rehabilitation, and patient satisfaction remain areas of ongoing investigation.

The primary objective of this study was to compare the short-term functional outcomes of ACLR using hamstring tendon and peroneus longus tendon autografts, with the Lysholm Knee Score at 12 weeks as the primary outcome measure. The secondary objective was to compare graft characteristics, including graft length and diameter, between the two groups. We hypothesized that the peroneus longus tendon autograft would provide favorable graft characteristics and improved early functional outcomes compared with the hamstring tendon autograft.

## Materials and methods

Study design and setting

This observational study was conducted in the Department of Orthopedics at Himalayan Institute of Medical Sciences, Swami Ram Nagar, Dehradun, over a period of 12 months (January 1, 2025 to December 31, 2025). Patients presenting with ACL injuries to the Orthopedics Department were screened for eligibility. Ethical clearance was obtained from the Institutional Ethics Committee (approval no. SRHU/HIMS/ETHICS/2026/360 dated October 19, 2024) before the start of the study, and written informed consent was obtained from all participants prior to enrollment.

Study population and sample size

The study included patients diagnosed with ACL tears who underwent primary ACLR using either hamstring tendon or peroneus longus tendon autografts. The primary outcome measure was the Lysholm Knee Score at 12 weeks postoperatively. Sample size was calculated using G*Power version 3.1 for the comparison of two independent means. Assuming a moderate effect size (Cohen’s d = 0.67), a two-sided significance level (α) of 0.05, and a study power of 80%, the minimum required sample size was 36 patients per group. To account for potential loss to follow-up, 40 patients were enrolled in each group, resulting in a total sample size of 80 participants. Consecutive sampling was used for patient recruitment. Patients were allocated to either the hamstring tendon or peroneus longus tendon graft group based on the operating surgeon’s clinical judgment after discussing both graft options with the patient. As this was an observational study, no randomization or predefined allocation protocol was used.

Selection criteria

Patients aged 18 years or older with ACL rupture requiring primary ACLR using either hamstring or peroneus longus tendon grafts were included in the study. Only patients who were able to ambulate normally and had no associated illness affecting mobility were considered eligible. Patients with immature skeletons, multiligament injuries, osteoarthritis of the knee, associated injuries requiring modification of rehabilitation protocol, or those requiring concurrent meniscal allograft, osteotomy, or major cartilage restoration procedures were excluded from the study.

Preoperative assessment

Baseline demographic details such as age, sex, and type of injury were recorded for all patients. Clinical and radiological evaluation was performed before surgery using the Lysholm Knee Scoring Scale [16], plain radiographs of both knees in anteroposterior and lateral views, and MRI of the affected knee. These investigations helped confirm the diagnosis and assess associated knee injuries.

Surgical procedure

**Figure 1:**
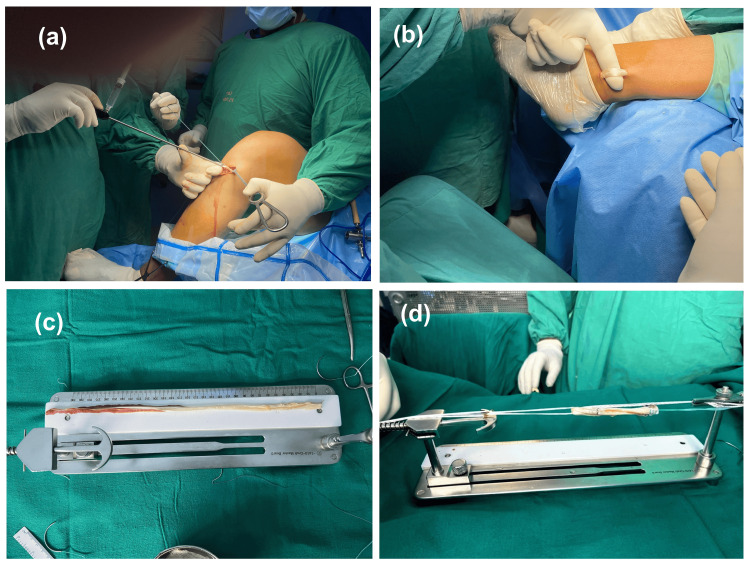
Intraoperative graft harvesting and preparation during ACLR. (a) Harvesting of the semitendinosus and gracilis (hamstring) tendon graft through an anteromedial proximal tibial incision. (b) Harvesting of the peroneus longus tendon graft through a posterolateral incision proximal to the lateral malleolus. (c) Preparation of the harvested tendon graft using whip stitches on a graft preparation board. (d) Final prepared graft mounted on the graft preparation station before arthroscopic passage and fixation. ACLR, anterior cruciate ligament reconstruction

All procedures were performed using a standardized arthroscopic technique with identical fixation methods. All procedures were performed by the same experienced orthopedic surgeon using a standardized surgical protocol. Patients underwent surgery under spinal anesthesia in the supine position with a pneumatic tourniquet applied to the thigh. Standard anteromedial and anterolateral arthroscopic portals were established, followed by diagnostic arthroscopy of the knee joint. Depending on the allocated graft, either the hamstring tendons (semitendinosus and gracilis) or the peroneus longus tendon was harvested. For the peroneus longus graft, a small longitudinal incision was made over the posterolateral aspect of the distal fibula, approximately 2-3 cm proximal to the lateral malleolus. The tendon was identified, harvested using a tendon stripper with care to protect the superficial peroneal nerve, and prepared with whip stitches to obtain the required graft length and diameter. For the hamstring graft, an oblique incision was made over the anteromedial aspect of the proximal tibia near the pes anserinus. The semitendinosus and gracilis tendons were identified, harvested using a tendon stripper, and similarly prepared with whip stitches. Arthroscopic ACLR was then performed using the same surgical technique in both groups. Anatomical femoral and tibial tunnels were created, the prepared graft was passed through the tunnels, and fixation was achieved with femoral fixation followed by tibial fixation using an interference screw (Figure 1).

Postoperative rehabilitation and follow-up

All patients followed the same standardized postoperative rehabilitation protocol. Patients were discharged on the sixth postoperative day with a knee immobilizer. Knee extension and ankle pump exercises were initiated immediately after surgery. Partial weight bearing with knee motion from 0° to 45° was permitted during the first two postoperative weeks. Full weight bearing was generally allowed after three to four weeks, and full knee flexion was usually achieved by five to six weeks. Functional outcomes were assessed using the Lysholm Knee Scoring Scale [16] preoperatively and at six and 12 weeks postoperatively. Ankle function was assessed clinically at each follow-up visit by evaluating ankle range of motion, plantar flexion and eversion muscle strength, and patient-reported symptoms. No validated ankle-specific functional scoring system was used.

Statistical analysis

Data were entered into Microsoft Excel (Microsoft Corporation, Redmond, WA, USA) and analyzed using IBM SPSS Statistics for Windows, version 26.0 (released 2018; IBM Corp., Armonk, NY, USA). Qualitative variables were expressed as frequencies and percentages, while quantitative variables were represented as mean ± SD. The chi-square test was used to analyze associations between categorical variables, and Student’s t-test was applied to compare continuous variables between groups. A p-value of less than 0.05 was considered statistically significant.

## Results

The mean age of patients in the hamstring group was 30.35 ± 8.58 years, while in the peroneus group it was 30.33 ± 9.35 years. The difference in mean age between the two groups was not statistically significant (t = -0.049, 95% CI: -4.203 to 4.003, p = 0.990), indicating that both groups were comparable with respect to age (Table 1).

**Table 1:** Baseline demographic and clinical characteristics of the study population.

Variable	Hamstring group (n = 40)	Peroneus group (n = 40)	Test statistic	95% CI	p-value
Age (years)	30.35 ± 8.58	30.33 ± 9.35	t = -0.049	-4.203 to 4.003	0.990
≤20 years	5 (12.5%)	7 (17.5%)	χ² = 2.680	-	0.612
21-30 years	17 (42.5%)	17 (42.5%)			
31-40 years	14 (35.0%)	11 (27.5%)			
41-50 years	3 (7.5%)	6 (15.0%)			
>50 years	1 (2.5%)	0 (0%)			
Male	30 (75.0%)	33 (82.5%)	χ² = 0.672	-	0.293
Female	10 (25.0%)	7 (17.5%)			
Weight (kg)	65.60 ± 11.46	68.03 ± 11.64	t = -0.939	-7.568 to 2.718	0.351
Height (cm)	169.25 ± 6.51	169.05 ± 5.37	t = 0.150	-2.457 to 2.857	0.881

Age-wise distribution showed that the majority of patients in both groups belonged to the 21-30 years age category, accounting for 17 (42.5%) patients each in the hamstring and peroneus groups. This was followed by the 31-40 years age group, which included 14 (35.0%) patients in the hamstring group and 11 (27.5%) patients in the peroneus group. Patients aged ≤20 years comprised five (12.5%) in the hamstring group and seven (17.5%) in the peroneus group, while those aged 41-50 years included three (7.5%) and six (15.0%) patients, respectively. Only one (2.5%) patient in the hamstring group was older than 50 years, whereas no patient in the peroneus group belonged to this age category. The age-group distribution between the two groups was comparable and did not show a statistically significant difference (χ² = 2.680, p = 0.612) (Table 1).

There was a male predominance in both groups. In the hamstring group, 30 (75.0%) patients were male, and 10 (25.0%) were female, whereas the peroneus group included 33 (82.5%) males and seven (17.5%) females. The difference in gender distribution between the groups was not statistically significant (χ² = 0.672, p = 0.293) (Table 1).

The mean body weight was 65.60 ± 11.46 kg in the hamstring group and 68.03 ± 11.64 kg in the peroneus group. The difference was not statistically significant (t = -0.939, 95% CI: -7.568 to 2.718, p = 0.351). Similarly, the mean height was 169.25 ± 6.51 cm in the hamstring group and 169.05 ± 5.37 cm in the peroneus group, with no statistically significant difference between the groups (t = 0.150, 95% CI: -2.457 to 2.857, p = 0.881). These findings indicate that both groups were demographically comparable at baseline (Table 1).

The mean final graft length in the hamstring group was 8.00 ± 0.58 cm, whereas the peroneus group demonstrated a significantly greater mean graft length of 8.37 ± 0.54 cm. The difference between the groups was statistically significant (t = -2.970, 95% CI: -0.626 to -0.124, p = 0.004). Similarly, the mean final graft diameter was 7.40 ± 0.46 mm in the hamstring group and 7.73 ± 0.57 mm in the peroneus group. The peroneus graft showed a significantly larger graft diameter compared to the hamstring graft (t = -2.869, 95% CI: -0.572 to -0.103, p = 0.005). These findings indicate that the peroneus longus tendon graft provided significantly greater graft length and diameter compared to the hamstring tendon graft (Table 2).

**Table 2:** Comparison of graft characteristics between the study groups.

Variable	Hamstring group (n = 40)	Peroneus group (n = 40)	Test statistic	95% CI	p-value
Final graft length (cm)	8.00 ± 0.58	8.37 ± 0.54	t = -2.970	-0.626 to -0.124	0.004
Final graft diameter (mm)	7.40 ± 0.46	7.73 ± 0.57	t = -2.869	-0.572 to -0.103	0.005

The mean preoperative Lysholm score was 64.95 ± 7.70 in the hamstring group and 66.40 ± 7.93 in the peroneus group. The difference between the groups was not statistically significant (t = -0.829, 95% CI: -4.933 to 2.033, p = 0.410), indicating that both groups had comparable baseline functional status before surgery. At six weeks postoperatively, the mean Lysholm score improved in both groups. However, the peroneus group demonstrated significantly higher functional scores (80.70 ± 5.98) compared to the hamstring group (74.90 ± 8.11). This difference was statistically significant (t = -3.639, 95% CI: -8.978 to -2.622, p = 0.001). At 12-week follow-up, further improvement in functional outcome was observed in both groups. The mean Lysholm score was significantly higher in the peroneus group (90.25 ± 3.46) compared to the hamstring group (84.57 ± 4.97), with the difference remaining statistically significant (t = -5.921, 95% CI: -7.587 to -3.763, p = 0.001). These findings suggest that although both grafts resulted in improved postoperative knee function, patients reconstructed with the peroneus longus tendon graft demonstrated superior short-term functional outcomes compared to those reconstructed with hamstring tendon grafts (Table 3).

**Table 3:** Comparison of Lysholm scores between the study groups.

Time interval	Hamstring group (n = 40)	Peroneus group (n = 40)	Test statistic	95% CI	p-value
Preoperative	64.95 ± 7.70	66.40 ± 7.93	t = -0.829	-4.933 to 2.033	0.410
Six weeks	74.90 ± 8.11	80.70 ± 5.98	t = -3.639	-8.978 to -2.622	0.001
12 weeks	84.57 ± 4.97	90.25 ± 3.46	t = -5.921	-7.587 to -3.763	0.001

In the preoperative period, most patients in both groups had either poor or fair Lysholm scores. Poor scores were observed in 15 (37.5%) patients in the hamstring group and 19 (47.5%) patients in the peroneus group, while fair scores were noted in 25 (62.5%) and 20 (50.0%) patients, respectively. No patient in the hamstring group had good or excellent scores preoperatively, whereas one (2.5%) patient in the peroneus group had an excellent score. The distribution of preoperative Lysholm score categories between the two groups was comparable and did not show a statistically significant difference (χ² = 2.026, p = 0.363) (Table 4).

**Table 4:** Distribution of Lysholm score categories at different follow-up intervals.

Time interval	Category	Hamstring group (n = 40)	Peroneus group (n = 40)	Test statistic	p-value
Preoperative	Poor	15 (37.5%)	19 (47.5%)	χ² = 2.026	0.363
	Fair	25 (62.5%)	20 (50.0%)		
	Good	0 (0%)	0 (0%)		
	Excellent	0 (0%)	1 (2.5%)		
Six weeks	Poor	5 (12.5%)	1 (2.5%)	χ² = 7.514	0.057
	Fair	31 (77.5%)	27 (67.5%)		
	Good	4 (10.0%)	10 (25.0%)		
	Excellent	0 (0%)	2 (5.0%)		
12 weeks	Poor	0 (0%)	0 (0%)	χ² = 19.17	0.001
	Fair	13 (32.5%)	0 (0%)		
	Good	24 (60.0%)	24 (60.0%)		
	Excellent	3 (7.5%)	16 (40.0%)		

At six weeks postoperatively, functional improvement was observed in both groups. In the hamstring group, five (12.5%) patients had poor scores, 31 (77.5%) had fair scores, and four (10.0%) had good scores, while no patient achieved an excellent score. In the peroneus group, poor scores were seen in only one (2.5%) patient, fair scores in 27 (67.5%) patients, good scores in 10 (25.0%) patients, and excellent scores in two (5.0%) patients. Although the peroneus group demonstrated a greater proportion of patients with good and excellent outcomes, the difference between the groups did not reach statistical significance (χ² = 7.514, p = 0.057) (Table 4).

At 12-week follow-up, a marked improvement in Lysholm score categories was observed in both groups, with significantly better outcomes in the peroneus group. In the hamstring group, 13 (32.5%) patients had fair scores, 24 (60.0%) had good scores, and three (7.5%) achieved excellent scores. In contrast, no patient in the peroneus group had a fair score, while 24 (60.0%) patients had good scores and 16 (40.0%) achieved excellent scores. The difference in distribution of Lysholm score categories between the groups at 12 weeks was statistically significant (χ² = 19.17, p = 0.001), indicating superior functional recovery in the peroneus longus graft group (Table 4).

Postoperative complications were minimal in both study groups. Knee stiffness was observed in one patient (2.5%) in the hamstring tendon graft group and one patient (2.5%) in the peroneus longus tendon graft group. No cases of surgical site infection, hamstring weakness, ankle weakness, graft failure, or persistent knee or ankle pain were reported in either group. Overall, both graft techniques demonstrated a low complication rate with no major postoperative adverse events, indicating comparable short-term safety profiles.

## Discussion

The present observational comparative study evaluated the functional outcomes of ACLR using hamstring tendon and peroneus longus tendon autografts. Both grafts demonstrated satisfactory postoperative recovery; however, patients reconstructed with the peroneus longus tendon showed superior short-term functional outcomes and better graft characteristics.

The demographic profile of patients in both groups was comparable. The mean age in the hamstring and peroneus longus groups was 30.35 ± 8.58 years and 30.33 ± 9.35 years, respectively, with most patients belonging to the 21-30-year age group. These findings are consistent with studies by Keyhani et al., Ertogrul et al., Khalil and Zawam, Teja et al., Khalid et al., and Vijay et al., all of whom reported comparable age distributions between graft groups and predominantly young adult populations undergoing ACLR [8,10,17-20]. ACL injuries are commonly seen in young, active individuals because of greater participation in sports and high-demand physical activities.

A male predominance was observed in both groups, with males accounting for 75.0% of the hamstring group and 82.5% of the peroneus longus group. Similar findings were reported by Keyhani et al., Ertogrul et al., Khalil and Zawam, and Khalid et al., where males constituted the majority of study participants [8,10,17,19]. This may reflect the greater involvement of males in physically demanding activities and contact sports associated with ACL injury.

Anthropometric parameters, including height and weight, were comparable between the two groups, indicating good baseline matching and minimizing confounding factors related to body habitus. Keyhani et al. similarly reported no significant difference in body composition between graft groups [8]. Comparable baseline characteristics strengthen the validity of postoperative outcome comparisons observed in the present study.

An important finding of the present study was the significantly greater graft length and graft diameter observed with the peroneus longus tendon. The mean graft length and diameter were significantly higher in the peroneus group compared to the hamstring group. These findings are in agreement with previous studies by Keyhani et al., Khalil and Zawam, Teja et al., and Khalid et al., all of whom demonstrated significantly larger graft diameters with the peroneus longus tendon [8,17-19]. Adequate graft diameter is clinically important because smaller grafts have been associated with increased graft failure rates and inferior biomechanical strength. The larger graft dimensions achieved with the peroneus longus tendon may therefore contribute to improved postoperative stability and functional outcomes.

Functional outcomes assessed using the Lysholm Knee Score improved significantly in both groups following surgery. Preoperative scores were comparable, indicating similar baseline knee function. However, patients in the peroneus longus group achieved significantly higher Lysholm scores at both six and 12 weeks postoperatively, suggesting better early postoperative functional recovery. Although these findings were statistically significant, they reflect outcomes during the early rehabilitation period and should not be interpreted as evidence of long-term graft superiority.

These findings partially differ from several previous studies that reported comparable long-term outcomes between grafts. Khalil and Zawam observed significant improvement in both groups at 18-month follow-up without a statistically significant intergroup difference [17]. Similarly, Teja et al. reported excellent functional outcomes in both groups with no significant long-term difference [18]. Khalid et al. also demonstrated significant postoperative improvement in both groups based on IKDC scores without intergroup significance [19]. Keyhani et al. and Ertogrul et al. likewise reported comparable long-term functional outcomes between the two grafts [8,10].

Despite these similarities, the present study demonstrated superior short-term outcomes in the peroneus longus group. Vijay et al. similarly observed slightly better postoperative functional scores with the peroneus longus tendon graft [20]. The better early outcomes seen in the present study may be related to preservation of hamstring muscle function when the peroneus tendon is used as the graft source. Hamstring preservation may facilitate earlier rehabilitation, improved knee flexor strength, and faster return of knee function during the early postoperative period.

The categorical distribution of Lysholm scores further supports this observation. At six weeks postoperatively, a greater proportion of patients in the peroneus longus group achieved good and excellent outcomes compared to the hamstring group. By 12 weeks, excellent outcomes were markedly more frequent in the peroneus group, while fair outcomes persisted only in the hamstring group. These findings suggest a faster transition toward higher functional categories with the peroneus longus graft and reinforce its advantage in early recovery.

Postoperative complications were minimal and comparable in both groups. No cases of surgical site infection, graft failure, persistent knee pain, or ankle pain were observed. Knee stiffness was noted in only one patient in each group. Importantly, no clinically significant ankle weakness or limitation of ankle range of motion was observed in the peroneus longus group. These findings support previous literature suggesting low donor-site morbidity associated with peroneus longus tendon harvest. Concerns regarding ankle instability remain an important consideration with peroneus tendon harvesting; however, no major functional deficit was identified in the present study during short-term follow-up.

The present study has several strengths. Both groups were managed within a single institution using similar surgical techniques, fixation methods, and rehabilitation protocols, ensuring procedural uniformity. Baseline demographic variables were comparable, minimizing selection bias. Functional outcomes were assessed using the validated Lysholm scoring system at predefined intervals, allowing objective comparison between grafts.

However, certain limitations should be acknowledged. First, the observational study design and relatively small sample size may limit the generalizability of the findings. Second, the 12-week follow-up reflects early postoperative rehabilitation and does not permit assessment of graft maturation, ligamentization, long-term graft survival, knee stability, or late donor-site morbidity. Therefore, the better functional outcomes observed in the peroneus longus group should be interpreted as improved early postoperative recovery rather than evidence of long-term graft superiority. Third, validated functional outcome measures such as the International Knee Documentation Committee (IKDC), Knee Injury and Osteoarthritis Outcome Score (KOOS), Tegner Activity Scale, return-to-sport assessment, Lachman test, pivot-shift test, instrumented laxity measurements, and objective muscle strength assessment were not performed. In addition, objective ankle assessment using validated tools such as the American Orthopaedic Foot and Ankle Society (AOFAS) Ankle-Hindfoot Score or Foot and Ankle Disability Index (FADI) was not performed. Finally, baseline variables including injury chronicity, time from injury to surgery, side involved, mechanism of injury, associated meniscal or chondral injuries, and pre-injury activity level were not evaluated and may have influenced postoperative outcomes. Delayed reconstruction may be associated with muscle wasting, secondary meniscal injury, and degenerative changes, which could have affected functional recovery. Future multicenter randomized controlled studies with larger sample sizes, comprehensive functional and ankle-specific assessments, and longer follow-up are required to validate these findings and establish the long-term efficacy and safety of the peroneus longus tendon autograft in ACLR.

## Conclusions

In the present study, the peroneus longus tendon autograft demonstrated significantly greater graft diameter and graft length than the hamstring tendon autograft, indicating favorable graft characteristics. Patients reconstructed with the peroneus longus tendon also achieved better early functional outcomes during the 12-week follow-up. However, these findings reflect early postoperative recovery and should not be interpreted as evidence of long-term graft superiority or ligamentization. Overall, the peroneus longus tendon appears to be a promising alternative autograft for ACLR. Nevertheless, larger randomized controlled studies with longer follow-up and comprehensive functional assessment are required to validate these findings and establish their long-term efficacy and safety.
